# Reports of the Transactions of the Chicago Medical Societies

**Published:** 1885-10

**Authors:** 


### The Reports of the Transactions of Chicago Medical Societies.

During the past year, the members of the various Chicago medical societies have at last awakened to the importance of correct reports of the transactions.

At the meeting of the Chicago Medical Society, held May 18th, 1885, resolutions were passed providing for a Committee on Publication. This Committee was authorized to secure the services of a stenographer, revise the short-hand report, and attend to the publication of the transactions.

DOCTOR E. J. DOERING and PROFESSOR D. W. GRAHAM were appointed members of this Committee, and DOCTOR J. A. ROBISON was chosen as the reporter. In order to secure faithful and complete reports of the transactions, the coöperation of the members participating in the discussions was solicited. The committee offered the following suggestions:

"I. Deliver to the reporter copies of all papers on the evening they are read.

"II. The reporter will hand each member a copy of the remarks, made by him during discussions, for his revision. This revision, to be published, must be returned to the reporter before noon of Thursday following the meeting of the society.

"III. The reporter, when necessarily absent, is authorized to have an alternate to perform his duties, who is entitled to the same recognition and coöperation as the reporter himself."

A very marked improvement in the character of the reports of the transactions of the CHICAGO MEDICAL SOCIETY, under the new *régime*, is apparent to the most casual observer.

A slightly different method has been adopted in the reports of the transactions of the CHICAGO GYNÆCOLOGICAL SOCIETY. Each Fellow, participating in a discussion, is requested to send a *résumé* of his remarks to the editor within the space of one week. Otherwise the remarks are omitted.

It is certainly very annoying to see one's remarks, in a discussion, emasculated, distorted beyond all recognition, published in a number of medical journals and widely circulated. Misrepresentation, as the result of the reporter's perversity or carelessness, is not at all infrequent. Under such circumstances, the reporter richly deserves censure. As a matter of fact, he is sure to come in for a full share of vituperation, whether merited or not. In all debates, there is much irrelevant talk. In medical discussions, the feature of prolixity and wandering from the subject is particularly prominent. Few medical gentlemen know how to express their ideas *clearly*, *distinctly* and *adequately*. On the contrary, their conceptions are usually *obscure* and *confused*.

MR. BAYNES has appended to his translation of the *Port Royal Logic* a complete translation of LEIBNITZ'S tract on the *necessary characters* of perfect knowledge. A careful study of the little tract by the members of any society would lessen the reporter's labors.

In the heat of discussion, the words of the debaters are not always characterized by accuracy of statement. A few months since, a distinguished gynecologist made the remarkable assertion that he had seen three cases of epithelioma of the *glans penis* result from copulation with women, whose crevices were the seat of carcinoma. A mortifying withdrawal of the remark followed, and the heat of discussion was urged in palliation.

The "accidence and syntax" of English grammar is constantly violated, and Dr. CAMPBELL'S canons are held in derision. Then there exists in the medical mind, a profound contempt for the "necessary laws of thought." Premises and conclusions, utterly at variance, are combined in a rambling, disconnected way, in total defiance of the laws of the syllogism. Fallacies,—logical and material,—exceed in kind and number those described by MR. JOHN STUART MILL. To bring order out of such chaos requires the continued exercise of a high degree of mental energy. Time, patience and attention are required to make the remarks clear, concise and relevant.

The plans adopted by the CHICAGO MEDICAL AND GYNÆCO-

LOGICAL SOCIETIES aredesigned to conciliate the members and secure a more creditable account of the proceedings.

They fail utterly to produce a truthful report of the transactions. On return home, the library is consulted in a perfectly calm mood, and a radically different tale is the usual resultant.

